# Editorial: Biomedical applications of natural polymers

**DOI:** 10.3389/fbioe.2022.1077823

**Published:** 2022-11-24

**Authors:** Qian Feng, Kunyu Zhang, Boguang Yang, Yongsheng Yu

**Affiliations:** ^1^ Chongqing Institute of Green and Intelligent Technology, Chinese Academy of Sciences, Chongqing, China; ^2^ Chongqing School, University of Chinese Academy of Sciences, Chongqing, China; ^3^ Bioengineering College, Chongqing University, Chongqing, China; ^4^ School of Biomedical Sciences and Engineering, South China University of Technology, Guangzhou, China; ^5^ Department of Biomedical Engineering, The Chinese University of Hong Kong, Hong kong, China

**Keywords:** biomedical applications, natural polymers, tissues repair, wearable sensor, hydrogel

## Introduction

Natural polymers are attracting a lot of interest for use in biomedical applications due to their high biocompatibilities and ease of modification. Regenerative medicine, drug delivery, and targeted therapy are the main biomedical applications for natural polymers. For biomedical applications, natural polymers are chosen for their biocompatibilities, porosities, hydrophobicities/hydrophilicities, surface energies, degradation rates, and other desirable characteristics. Moreover, they can undergo a diverse range of chemical or physical modifications to fulfill the requirements for specific biomedical applications.

This Research Topic is designed to attract recent, novel findings in regenerative medicine, peptide/protein modulators of protein-protein interactions, natural polymer-based biosensors in biomedical applications, natural polymers for drug delivery, computer-aided design of original natural polymers, design and preparation of antibiosis natural polymers, and functionalization of virus-like particles. The Research Topic incudes 14 high-quality papers focused on the research areas highlighted above.

## Applications in tissue repair

Nature polymers are frequently used to construct scaffolds with customized structures and functionalities that can improve cell growth and the formation of new tissue. Zheng et al. reported a linearly cross-linked sodium HA hydrogel (HA-engineered hydrogel) used as a retinal patch in the rabbit rhegmatogenous retinal detachment (RRD) model. The HA-engineered hydrogel exhibited a similar dynamic viscosity, cohesiveness, and G′ compared with the commercial HA hydrogel. The findings demonstrated that the HA-engineered hydrogel can facilitate complete retinal reattachment without the need for silicone oil endotamponade or expansile gas. It may serve as a promising retinal patch for sealing retinal breaks during retinal detachment repair.

By cross-linking GO-arabinoxylan and polyvinyl alcohol (PVA) with tetraethyl orthosilicate (TEOS), Ul-Haq and coworkers functionalized arabinoxylan and graphene oxide (GO) using a hydrothermal method to produce multifunctional composite hydrogels. The hydrogel accelerated wound healing and promoted vascularization, with no major inflammation observed within 7 days. In order to improve the efficiency of ginsenosides (GS) transdermal absorption, Jin et al. prepared delivery vehicles using GS liposomes (GSLs) and GS niosomes (GSNs). The vehicles suppressed skin lipid peroxidation caused by ultraviolet (UV) radiation and reduced the amounts of MMPs and inflammatory cytokines in skin tissue.

Chen et al. summarized recent progress in natural polymer-based scaffolds for soft tissue repair. Furthermore, the authors discussed challenges in clinical translations and materials design. Zhang et al. reviewed the physicochemical properties and the latest applications of hydrogels in premature ovarian failure and intrauterine adhesion. The authors also summarized the limitations in clinical application of hydrogels and provide future prospects. Yan et al. proved that human umbilical cord mesenchymal stem cell-derived exosomes can accelerate diabetic cutaneous wound healing, providing a promising therapeutic strategy for chronic diabetic wound repair. Yang et al. reviewed the various structures of natural polysaccharides with high commercial values, and their various applications in treating various oral diseases such as drug delivery, tissue regeneration, material modification, and tissue repair.

## Applications as wearable sensors

Owing to the advantages of hydrogels, hydrogel-based flexible electronic devices were developed for future healthcare and biomedical applications. Chen et al. designed a mechanically resilient and conductive hydrogel exhibiting a double-network structure. The first dense network comprised Ca^2+^-crosslinked alginate, and the second loose network consisted of ionic pair-crosslinked polyzwitterion. The results demonstrated the enduring accuracy and sensitivity of the hydrogel in detecting human motions, including large joint flexion, foot planter pressure measurement, and local muscle movement. Hu et al. developed a natural polymer-based conductive hydrogel formed by the Schiff base reaction between hydrazide-grafted hyaluronic acid and oxidized chitosan, with added KCl employed as a conductive filler. The hydrogel exhibited excellent mechanical properties, good sensitivity (GF = 2.64), durability, and stability, even in cold conditions (−37°C).

## Applications in other fields

There is no effective treatment for placental dysfunction. Therefore, Jiang et al. reviewed nanotechnologies for placental dysfunctional. In order to provide a foundational understanding of placental dysfunction, potential delivery targets, and recent research on placenta-targeted nanoparticle delivery systems for the potential treatment of placental dysfunction, the authors highlighted candidate nanoparticle-loaded molecules. Xia et al. summarized the structures and biological characteristics of chitosan and its derivatives. Moreover, the authors reviewed their applications in therapeutics, drug delivery, anti-infection, wound healing, tissue regeneration, and anticancer. Although absorbable plates and screws are used to treat rib fractures in clinical settings, it is unclear which type of screw fixation method is more effective. Thus, Xue et al. evaluated five different types of screw fixation methods on anterior ribs, lateral ribs, and posterior ribs, using finite element analysis. The authors provided a basis and a reference for clinical application, and presented the best screw fixation method on an absorbable plate for rib fractures. Chen et al. developed an injectable hyaluronic acid (HA)/oxidized chitosan (OCS) hydrogel that slowly released micro hypochlorous acid (HClO). The positive charge of OCS can introduce a sustainable antibacterial effect. This hydrogel may be a promising wound dressing material in clinical treatments.

## Outlook

Natural polymers have been broadly utilized in tissue culture, wound treatment, implantation, controlled drug delivery, targeted therapy of diseases, etc. However, their expansion in biomedical applications has encountered two main challenges: 1) limited strategies for functional modification of natural polymers, and 2) limited new fields of application. Fourteen top-quality articles have been published in this Research Topic on biomedical applications of natural polymers. We hope that this Research Topic proves meaningful for novel natural polymer designs, the evolution of advanced fabrication techniques, and biomedical applications.

